# Mass Die-Off of Saiga Antelopes, Kazakhstan, 2015

**DOI:** 10.3201/eid2506.180990

**Published:** 2019-06

**Authors:** Sasan Fereidouni, Graham L. Freimanis, Mukhit Orynbayev, Paolo Ribeca, John Flannery, Donald P. King, Steffen Zuther, Martin Beer, Dirk Höper, Aidyn Kydyrmanov, Kobey Karamendin, Richard Kock

**Affiliations:** University of Veterinary Medicine Vienna, Vienna, Austria (S. Fereidouni); The Pirbright Institute, Pirbright, UK (G.L. Freimanis, P. Ribeca, J. Flannery, D.P. King);; Research Institute for Biological Safety Problems, Otar, Kazakhstan (M. Orynbayev);; Association for the Conservation of Biodiversity of Kazakhstan, Astana, Kazakhstan (S. Zuther);; Frankfurt Zoological Society, Frankfurt, Germany (S. Zuther);; Friedrich-Loeffler-Institut, Greifswald-Insel Riems, Germany (M. Beer, D. Höper);; Institute of Microbiology and Virology, Almaty, Kazakhstan (A. Kydrymanov, K. Karamendin);; Royal Veterinary College, London, UK (R. Kock)

**Keywords:** Pasteurella multocida, outbreaks, Kazakhstan, Pasteurella infections, saiga, antelopes, high-throughput sequencing, HTS, metagenomics, mass mortality, die-off, bacteria

## Abstract

In 2015, a mass die-off of ≈200,000 saiga antelopes in central Kazakhstan was caused by hemorrhagic septicemia attributable to the bacterium *Pasteurella multocida* serotype B. Previous analyses have indicated that environmental triggers associated with weather conditions, specifically air moisture and temperature in the region of the saiga antelope calving during the 10-day period running up to the event, were critical to the proliferation of latent bacteria and were comparable to conditions accompanying historically similar die-offs in the same areas. We investigated whether additional viral or bacterial pathogens could be detected in samples from affected animals using 3 different high-throughput sequencing approaches. We did not identify pathogens associated with commensal bacterial opportunisms in blood, kidney, or lung samples and thus concluded that *P. multocida* serotype B was the primary cause of the disease.

The saiga antelope (*Saiga tatarica tatarica* and *S.t. mongolica*) is a critically endangered species (*1*) with populations located in Kazakhstan in addition to small remnants in Russia and Uzbekistan and a subspecies in Mongolia. Each year during the month of May, Saiga antelopes gather in Kazakhstan for calving. Mass die-offs in their populations have been reported previously and were attributed to viral and bacterial etiologies, including pasteurellosis (*2*). However, the diagnosis in most of these events has been unreliable because of insufficient fresh sampling and diagnostic work (*2*).

During a large outbreak in 2015, extensive diagnostics and environmental studies were undertaken, subject to restricting factors such as remoteness and limited cold chain resources. Annual disease monitoring in saiga antelopes had been established after die-offs occurred in western Kazakhstan in 2010, and an international multidisciplinary research team was on the ground at the time of the die-off, performing routine surveillance (*3**,**4*).

The mass die-off of saiga antelopes in Kazakhstan started around May 10, 2015, and caused ≈200,000 deaths across several calving groups within 3 weeks. These subgroups of saiga antelopes were spread discretely across a landscape of several hundreds of thousands of square kilometers. The number of dead animals constituted more than two thirds of the global population of saiga antelope at the time. The outbreak wiped out 88% of the Betpak-Dala population in central Kazakhstan (*5*) and appeared to have a 100% case-fatality rate.

Laboratory results on the microbiologic, pathologic, and environmental conditions at the time of the 2015 outbreak suggested hemorrhagic septicemia caused by *Pasteurella multocida* serotype B and triggered by environmental conditions (*3*,*6*). However, whether a second unknown infectious agent had predisposed the animals to infection with *P. multocida* was unclear from the laboratory results. Given the opportunistic nature of *Pasteurella*, the objective of our study was to attempt to identify whether any additional unknown potential causative pathogens were present in samples (taken from 10 animals) that might may have contributed to the die-off.

## Materials and Methods

### Field Assessment

The first dead animals were detected in the Amangeldy District (Kostanay region) of Kazakhstan on May 10, 2015, and additional die-offs were recorded in unconnected discrete locations in the Aktobe and Akmola regions (*3*). A primary diagnosis of hemorrhagic septicemia as the cause of death was proposed at the sites on the basis of clinical signs and gross pathology. We took FTA papers of whole blood spots from 8 freshly dead, female animals (Table 1) in a 2-km radius on the last 2 days of the operation and sent them to international reference laboratories for high-throughput sequencing (HTS) protocols. FTA cards were used as backup given the limited resources available and difficulties in maintaining cold chain and in transportation of fresh samples to local laboratories. Lung and kidney tissue from 2 dead saiga antelopes (lung tissue from animal X and kidney tissue from animal Y) from the Turgai River region were also processed for 16S metagenomics sequencing in the city of Almaty, Kazakhstan. Although these samples were from a region 175 km from the site where the FTA card samples were taken, they were considered part of the same saiga antelope population. Given the uniformity of the clinical syndrome and consistency of the pathogenesis, the sample of cases selected was small relative to the scale of the die-off, but each case was evaluated in considerable depth and considered representative of the affected population on the basis of the consistent pathology and disease characteristics observed in all the affected animals (*3*).

**Table 1:** Details outlining the origins of the 8 FTA samples, including animals and GPS data, used in an investigation of a mass die-off of saiga antelopes, Kazakhstan, 2015*

Sample no.	Species	Age, y/sex	Comment	Sample type	GPS no.	Date
1	*Saiga tatarica*	3–4/F	Postmortem	FTA x2	427	2015 May 26
2	*Saiga tatarica*	3/F	Postmortem	FTA x2	426	2015 May 26
3	*Saiga tatarica*	1–2/F	Postmortem	FTA x2	456	2015 May 26
4	*Saiga tatarica*	1–2/F	Postmortem	FTA x2	452	2015 May 26
5	*Saiga tatarica*	5–6/F	Postmortem	FTA x2	457	2015 May 26
6	*Saiga tatarica*	>5/F	Postmortem	FTA x2	458	2015 May 26
7	*Saiga tatarica*	2/F	Postmortem	FTA x2	455	2015 May 26
8	*Saiga tatarica*	13/F	Postmortem	FTA x2	NA	2015 Jun 25

*GPS, global positioning system; NA, no information available.

### Laboratory Assessment

**Figure 1:**
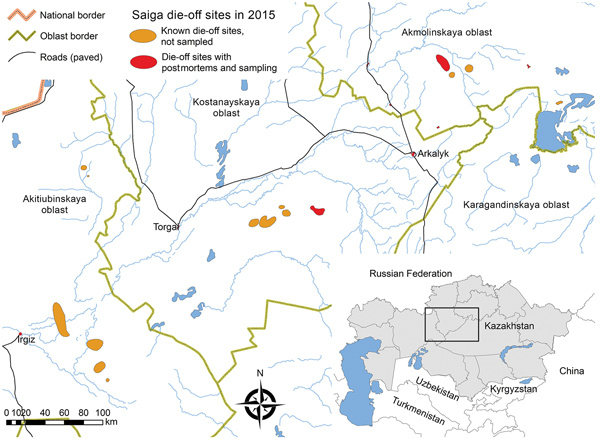
Geographic distribution of saiga antelope die-off events, Kazakhstan, 2015. Red and orange areas indicate known outbreak locations of the 3 saiga populations. Inset shows area in relation to the rest of Kazakhstan and neighboring countries of central Asia.

**Figure 2:**
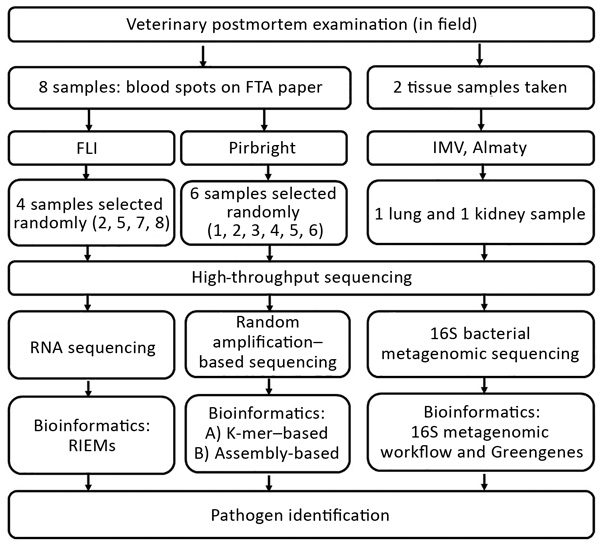
Outline of the process of sampling and high-throughput sequencing protocols performed at 3 research institutes in an investigation of a mass die-off of saiga antelopes, Kazakhstan, 2015. FLI, Friedrich-Loeffler-Institut; IMV, Institute of Microbiology and Virology.

We submitted samples of dried blood spots (2 cm in diameter) on FTA papers taken from 8 animals to 2 different research institutions (the Pirbright Institute in the United Kingdom and the Friedrich-Loeffler-Institut [FLI] in Germany) for HTS analyses (Figure 1) under 2 different HTS protocols (random amplification–based sequencing at Pirbright and RNA sequencing at FLI). Six of the 8 FTA blood spot samples were processed for further testing by using HTS at Pirbright, and 4 of the 8 samples were processed for further testing at FLI. Two of the 8 samples were processed by both laboratories. Lung and kidney tissue from 2 dead saiga antelopes (Table 2) were tested for 16S bacterial diversity by using a 16S metagenomic sequencing protocol developed by the Institute of Microbiology and Virology in Almaty (Figure 2).

**Table 2:** Characteristics of fresh tissue samples transferred to Almaty for 16S ribosomal profiling used in an investigation of a mass die-off of saiga antelopes, Kazakhstan, 2015*

Animal	Date	GPS	Species	Age y/sex	Sample used for HTS
Animal X	2015 May 16	49°46.586N/ 65°26.369E	*Saiga tatarica*	2/F	Lung
Animal Y	2015 May 19	49°45.001N/065°27.536E	*S. tatarica*	3/F	Kidney

*HTS, high-throughput sequencing.

## Results

We analyzed reads from each of the parallel investigations by using established bioinformatics pipelines to identify microbial agents present within each sample. All raw datasets, de novo assemblies, and 16S sequencing metagenome datasets have been submitted to the European Nucleotide Archive and GenBank (accession nos. PRJEB28164, PRJEB28184, and PRJNA486600).

### Random Amplification–Based Sequencing Protocol

The Pirbright protocol consisted of a random amplification workflow, with libraries sequenced using the MiSeq System (Illumnia, https://www.illumina.com) to identify microbial nucleic acids present in dried peripheral blood spots. The classification of sequenced reads into different taxonomic groups was conducted by using 2 approaches; the first was a k-mer–based approach that assigned each read independently (Table 3; Appendix Table 1), and the second was a de novo approach that first assembled reads into contigs and then assigned contigs (Table 4; Appendix Tables 2–4). Approximately 72% of the original reads mapped to the assembly produced by the de novo approach. Neither of these 2 approaches conclusively identified a single virus as a causative agent in all samples. In all 6 samples tested, 46.4% (geometric mean [GM]) of reads were unclassified against the Mini-Kraken Database (https://ccb.jhu.edu/software/kraken) and were possibly host-derived; these reads were labeled as unclassified (Table 3). This determination was further supported by the de novo analysis, which identified the largest contigs as being host-derived, having 35% of reads accounting for host material. In terms of microbial organisms, in 6 of 6 samples, the largest numbers of hits (GM 39%) were identified as *Pasteurella* spp. The specificity of this finding was increased for the *P. multocida* genome, which exhibited the greatest number of matches (GM 69,760 hits/sample [35.4%]). The microbial organism with the second highest number of hits in all samples was *Alteromonas macleodii* (GM 215 hits/sample [0.11%]). K-mers present in all 6 samples also aligned with the *Achromobacter xylosoxidans* (GM 102 hits/sample), *Haemophilus* spp. (GM 40 hits/sample), *Mannheimia haemolytica* (GM 13 hits/sample), *Klebsiella* spp. (GM 23 hits/sample), and *Aggregatibacter* spp. (GM 16 hits/sample), albeit to a lower level than the top 2 results. All the other organisms tested had matches of <10 hits and were not considered statistically significant.

**Table 3:** Main results of the k-mer–based approach on the random amplification metatranscriptomic dataset used in an investigation of a mass die-off of saiga antelopes, Kazakhstan, 2015*

Organism	No. reads (% total reads)					
	Sample 1	Sample 2	Sample 3	Sample 4	Sample 5	Sample 6
Total no. reads	231,907	773,835	272,102	300,807	235,255	187,049
Total no. reads passing QC	109,302 (47.1)	553,163 (71.48)	174,613 (64.17)	233,888 (77.8)	171,409 (72.86)	138,292 (73.93)
Unclassified/nonmicrobial	50,478 (46.18)	343,404 (62.08)	86,165 (49.35)	133,456 (57.06)	57,812 (33.73)	50,731 (36.68)
Virus	47 (0.03)	141 (0.03)	33 (0.03)	174 (0.05)	73 (0.04)	51 (0.04)
*Pasteurellaceae*	53,097 (48.58)	129,337 (23.38)	69,251 (39.66)	63,817 (27.29)	93,378 (54.48)	72,799 (52.64)
*Pasteurella multocida*	49,844 (45.6)	115,231 (20.83)	60,504 (34.65)	56,775 (24.27)	86,664 (50.56)	67,406 (48.74)
*Alteromondales*	1303 (1.19)	2690 (0.49)	35 (0.02)	499 (0.21)	32 (0.02)	51 (0.04)
*Enterobacteriaceae*	52 (0.05)	208 (0.04)	153 (0.09)	112 (0.05)	77 (0.04)	48 (0.03)
*Haemophilus*	23 (0.02)	77 (0.01)	31 (0.02)	36 (0.02)	63 (0.04)	37 (0.03)
*Betaproteobacteria*	86 (0.08)	13160 (2.38)	30 (0.02)	86 (0.04)	18 (0.01)	22 (0.02)
*Mannheimia*	10 (0.0)	18 (0.0)	12 (0.01)	18 (0.01)	19 (0.01)	9 (0.01)
*Aggregatibacter*	8 (0.0)	37 (0.0)	13 (0.0)	18 (0.0)	16 (0.0)	18 (0.01)
*Klebsiella*	11 (0.0)	58 (0.01)	16 (0.0)	54 (0.01)	16 (0.0)	14 (0.0)

*Only organisms that were identified in all samples and with >10 reads are listed. Samples 2 and 5 were also tested at Friedrich-Loeffler-Institut by using an RNA sequencing protocol. QC, quality control.

**Table 4:** Main results obtained using a de novo approach on the random amplification meta-transcriptomic dataset used in an investigation of a mass die-off of saiga antelopes, Kazakhstan, 2015*

Read area	No. contigs†	Total length, bp	Attribution‡	Comment
796	2	271	*Pasteurella bettyae* CCUG 2042	
1,758,115	162	27,999	*Ovis canadensis canadensis*	Host
1,676,355	23	5,780	*Capra hircus* (goat)	
36,795	7	1,287	*Bubalus bubalis* (water buffalo)	
30,763	14	1,959	*Bos taurus* (cattle)	
3,252	6	1,366	*Saiga tatarica*	
2,625	8	1,283	*Ovis aries* (sheep)	
2,414	5	969	*Bos indicus*	
1,650	2	317	*Eudorcas thomsonii* (Thomson’s gazelle)	
14,221,307	6,641	2,103,430	*Pasteurella multocida*	Other
69,009	195	27036	Unknown sequence	
35,246	1	401	Uncultured eukaryote	
796	2	271	*Pasteurella bettyae* CCUG 2042	

*Equivalent to the contig length × the average read coverage. †Number of contigs with the same attribution. ‡As determined by the best blastn hit.

The de novo analysis approach also did not identify any homologies with unexpected viral genomes. Several bacteria were identified by both k-mer and de novo analysis protocols, including *P. multocida* and *M. haemolytica*, although these bacteria had smaller contigs (182 bp and 85 bp, respectively) and were thus not included in the results (Table 4). We were unable to identify 195 contigs produced by the de novo assembly despite using several BLAST databases (https://blast.ncbi.nlm.nih.gov/Blast.cgi). We subjected these contigs to an extended analysis in which they were first aligned to BLAST databases with tblastx to find similarities at the protein level. That analysis generated matches for 35 contigs; the distribution of the matches in terms of species mirrors quite closely the one found by nucleotide BLAST (Appendix Tables 2–4). Separately, we also translated and subjected the unknown contigs to a search using SUPERFAMILY (http://supfam.org). This approach returned hits for 87 contigs (Appendix Tables 2–4), of which most appeared to be homologs of bacterial proteins. No further pathogens could be conclusively identified using this analysis. Further analysis of the assembled sequences attributed to *P. multocida* did not permit accurate conclusions to be drawn because of the fragmented nature of the contigs.

### RNA Sequencing Protocol

By using the RIEMS analysis pipeline, we performed taxonomic analysis of the sequencing reads obtained from libraries generated from RNA extracted from 4 blood spots from FTA cards that had been transcribed into cDNA using random hexamer priming followed by shotgun library preparation. Of the samples tested at FLI, samples 2 and 5 were also tested at Pirbright. Overall, these analyses classified 77.9%–93.5% of the reads as *P. multocida* (Table 5). The remainder mainly represented host sequences (0.9%–16.2%). With a few exceptions for phage reads, no reads were classified as being of viral origin, which was concordant with findings of the Pirbright dataset. In all samples, the proportion of reads remaining unclassified after analysis of the nucleic acid sequences was low (0.42%–0.45%); these unclassified reads had median lengths of 35 bp (interquartile range 25–45 bp) and accounted for 0.068%–0.082% of the total bases. Therefore, the information content of the unclassified portion of the datasets was too low to provide additional information even by additional analyses on the basis of the amino acid sequences deduced from these reads.

**Table 5:** Summary of the most relevant results obtained by RIEMS analyses of the datasets (sequenced from shotgun libraries generated from random primed cDNA) used in an investigation of a mass die-off of saiga antelopes, Kazakhstan, 2015

Organism	No. (%) reads			
	Sample 2* (lib01416)	Sample 5* (lib01417)	Sample 7 (lib01418)	Sample 8 (lib01419)
Input reads	411,640 (100)	376,210 (100)	372,387 (100)	354,958 (100)
Quality filtered reads†	12,786 (3.1)	10,793 (2.9)	11,559 (3.1)	10,895 (3.1)
Unclassified reads†	1,776 (0.43)	1,520 (0.40)	1,626 (0.44)	1,494 (0.42)
				
Classified reads†	397,078 (96.5)	363,897 (96.7)	359,202 (96.5)	342,569 (96.5)
Host‡	64,618 (16.3)	4,770 (1.3)	3,414 (1.0)	4,784 (1.4)
*Pasteurellaceae*‡	317,009 (79.8)	345,893 (95.1)	339,484 (94.5)	324,770 (94.8)

*Samples 2 and 5 were also tested by using high-throughput sequencing at the Pirbright Institute. †Percentage is of the number of input reads. ‡Percentage is of the number of classified reads.

To conduct a detailed analysis of the numerous *P. multocida* organisms detected, we mapped the complete datasets along the *P. multocida* genome sequence (GenBank accession no. NC_002663.1). We then performed blastx (*7*) analyses of the resulting contigs for a basic function prediction of the expressed genes. Besides detecting genes encoding proteins of gene expression, general metabolism, and cell division, these analyses detected several proteins associated with pathogenicity. For example, proteins facilitating active iron uptake (iron ABC transporter permease [GenBank accession no. WP_010906625], iron ABC transporter substrate binding protein [accession no. WP_005715971.1], iron binding protein [accession no. WP_005726096.1], and iron permease [accession no. WP_010906655.1]) or proteins of the oxidative stress response (catalase [accession no. WP_010906440], superoxide dismutase [accession no. WP_005750998], peroxiredoxin [accession no. WP_005716614.1]). These analyses also revealed expression of genes encoding stress- and starvation-induced proteins (stringent starvation protein A homologue [accession no. WP_005726291.1]) and the virulence factor SrfB (accession no. WP_005755436.1).

### 16S Metagenomic Sequencing Protocol

We applied a metagenomics workflow for classifying organisms from the V3 and V4 regions of the 16S rRNA gene by using a Greengenes database (http://greengenes.lbl.gov) to test tissue taken from 2 animals (lung tissue from animal X and kidney tissue from animal Y) (Table 6). Among the variable regions of 16S gene, V3 is a highly variable region that can distinguish bacteria to the genus level. V4 is also efficient but less so than V3 (*8*). The output of the workflow classified the reads at the primary taxonomic levels (kingdom, phylum, class, order, family, genus, and species).

**Table 6:** Top 8 of 94 species classification results after 16S bacterial metagenome sequencing in an investigation of a mass die-off of saiga antelopes, Kazakhstan, 2015*

Classification	No. reads			% Total reads	
	Lung (animal X)	Kidney (animal Y)		Lung (animal X)	Kidney (animal Y)
*Pasteurella multocida*	25,625	6,907		44.06	48.32
Unclassified at species level	21,246	4,990		36.53	34.91
*Pasteurellaceae*	7,101	1,536		12.21	10.75
*Pasteurella pneumotropica*	3,298	580		5.67	4.06
*Mannheimia caviae*	462	78		0.79	0.55
*Serratia entomophila*	50	17		0.09	0.12
*Bacillus horneckiae*	49	16		0.08	0.11
*Vagococcus teuberi*	39	13		0.07	0.09
*Sporolactobacillus putidus*	50	0		0.09	0
*Acinetobacter gerneri*	49	0		0.08	0
*Gallibacterium melopsittaci*	39	0		0.07	0

*Total species-level taxonomic categories identified: 94 for lung sample (animal X) and 68 for kidney sample (animal Y).

Sequencing statistics revealed the number of total reads to be 63,508 for lung tissue and 15,422 for kidney tissue. The number of reads passing quality filtering was 58,161 for lung tissue and 14,291 for kidney tissue. The percentage of reads passing quality filtering was 91.6% for lung tissue and 92.7% for kidney tissue.

Of all reads generated, 86.80%–89.05% of all short reads were from bacteria of the genus *Pasteurella*, of which 44.06%–48.32% were identified as *P. multocida*. Other species were *Pasteurellaceae* (10.75%–12.21%), *P. pneumotropica* (4.06%–5.67%), and those unclassified at species level (34.91%–36.53%) (Table 5). More than 80% of unclassified reads at the species level belonged to the *Pasteurella* genus.

## Discussion

Saiga antelopes are a critically endangered species (*1*), and the population is increasingly fragmented and vulnerable to stochastic events such as disease epidemics. The mass die-off in Kazakhstan and the small population of ≈10,000 in Mongolia recently devastated by peste des petits ruminants (PPR) virus in 2017 illustrates this point (*3*). The saiga antelopes undertake large-scale seasonal migrations between their summer and winter ranges because of the extreme variation in climate conditions and the need for pastures offering sufficient forage. The calving sites are highly variable from year to year and depend on plant phenology, environmental factors, and anthropogenic effects (*9*). The analysis of available data showed that the number of saiga antelopes in Kazakhstan over the past 60 years has fluctuated widely, from ≈2 million in the 1970s to ≈50,000 animals in the early 21st century because of poaching and other factors, including a series of mass die-offs (*10*,*11*). A few incidences of infectious disease, including foot-and-mouth disease, have been confirmed (*12*), but most events were attributable to pasteurellosis; *M. haemolytica* and *P. multocida* were isolated on occasion (*13*). However, diagnoses are lacking comprehensive clinical, pathological, epidemiologic, and environmental investigation and remain tentative in all cases outside the 2015 event. Diagnosis of wildlife deaths is constrained by the fact these populations are not managed nor always monitored regularly, meaning die-offs occur frequently and investigators often do not have access to fresh carcasses. In the 2015 saiga antelope event, a monitoring team was in place in 2 of the 15 die-off locations and were equipped for general diagnostic work. This situation was unusual and provided a unique opportunity, but the unpredictability of such an event happening limited the extent of the outbreak investigation. Sampling was necessarily strategic, and because all of the animals in the population were affected by the same syndrome and died, the sample size did not need to be large or statistically representative. Each case would have an equal chance of providing the result, and failure to diagnose would be more likely a product of insufficient material per case or loss of viability of organisms because of cold chain and storage issues.

Nevertheless, the findings obtained from this work are representative of the population for a few reasons. First, the clinical syndrome was uniform in both the adult and calf populations. We observed no statistically significant variation in the temporal progression once symptoms were noted, and clinical signs and gross pathology were highly consistent. Second, the 100% mortality rate among the herds indicates a universal effect, and no samples taken were likely to be nonrepresentative or attributable to an alternate etiology. In addition, the rapidity of the syndrome precluded large numbers of cases being investigated by our relatively small team because necropsy and sampling for each case took several hours to complete.

Microbiologic and virologic diagnostic methods showed that in samples from >90% of saiga antelopes that died in 2015, the cause was pasteurellosis (*3*,*6*). Previous studies had demonstrated the absence of other potential causative agents by diagnostic PCR, including bacteria (e.g., anthrax bacillus, *Coxiella burnetti*, *Erysipelothrix rhusiopathiae*, and *Listeria* spp.), mycoplasma (e.g., *Mycoplasma ovipneumoniae*), and virus diseases (e.g., foot-and-mouth disease, bluetongue, PPR, epizootic hemorrhagic disease, sheep pox, Akabane, Aujesky’s disease, bovine viral diarrhea, visna-maedi, and malignant catarrhal fever) (*3*,*6*). Furthermore, these studies used capsular typing with specific primers to show that strains of *P. multocida* from saiga antelopes belonged to serogroup B (*3*,*6*). Our 16S analysis also showed that the *P. multocida* isolated from saiga antelopes in 2015 in Akmola and Kostanay oblasts were genetically identical to the bacteria isolated from saiga antelopes in 1988, 2010, 2011, and 2012, as well as *P. multocida* subsp*.multocida*_PM30 strain (GenBank accession no. AY299312) isolated from ill cattle with hemorrhagic septicemia in 2004 (*6*).

Three different HTS protocols were used in parallel to identify unknown microbial pathogens that played a potential role in disease pathogenesis: a protocol based on random amplification using Illumina sequencing; an RNA-based analysis without amplification combined with sequencing on an Ion Torrent (ThermoFisher, https://www.thermofisher.com); and a bacterial 16S sequencing pipeline using Illumina technology. Each of these workflows demonstrated the potential for different experimental challenges in obtaining metagenomic datasets (e.g., biases in amplification-based protocols and the use of low-input starting material in no-amplification protocols) (*14*). The high sensitivity of such methods to detect small amounts of nucleic acids also poses challenges in terms of prevention of contamination and false-positive results. Caution should be exercised in drawing conclusions from such datasets without appropriate validation. In addition to blood spots, other tests, including bacteriologic and virologic tests on various tissues and samples taken, were conducted locally in Kazakhstan at government laboratories and reported elsewhere (*3*).

Despite the high sensitivity of the methodologies we used, our study is somewhat limited by the sample type (FTA cards), which precludes the detection of pathogens in lymphoid tissues and other organs. The use of FTA cards might also introduce biases in the testing protocols, which can favor or hinder the detection of certain types of viruses (*15*).

Both metagenomic protocols conclusively identified *Pasteurella* spp. in large numbers of reads compared with other pathogens; these findings were then confirmed in a third pipeline using 16S bacterial ribosomal RNA sequencing. Further analysis of *P. multocida* bacterial sequences suggested that the expression of metabolic- and stress-related proteins might suggest that the bacteria were actively growing and in active competition with the host organism for essential nutrients, especially iron, as shown by the expression of the genes coding for iron uptake systems.

Our de novo assembly approach also identified 195 short contigs that could not be attributed to any sequence present in several BLAST databases; of those, only 47 were identified using tblastx. The subsequent analysis based on SUPERFAMILY was only able to find protein homologies for 6 contigs, with most of them having homologies to bacterial genes. Whether this result is important is unclear; our unknown contigs might belong to >1 uncultured bacteria that have not been sequenced before. Previously published metagenomic studies have resulted in as many as 50% unidentifiable reads (*16*,*17*); the figures for our work are reduced in comparison, in particular when considering the results of our de novo approach (≈72% of our reads map to our assembly and <1% of the reads map to the contigs that we are unable to identify) (Appendix Table 2). Overall, the amount of unexplained sequence seems relatively small, in particular when considering the substantial number of species of bacterial, viral, and eukaryotic genome that remain either to be discovered or characterized. The simple fact that not all organisms have been sequenced or are available on central sequence repositories will always contribute to a percentage of unidentifiable reads.

*P. multocida* is a ubiquitous organism, most probably widely present in the saiga antelope population in its latent form. The potential pathogenicity is inherent in the organism and can be triggered opportunistically at any time in response to environmental triggers. The epidemiology of and observations on the spatiotemporal distribution of ill animals and carcasses in this study suggests that transmission of bacteria from animal to animal did not occur in most cases (except from mothers to calves, which occurred through infected milk). The near synchronous events in discrete subpopulations, with large distances between aggregations of many hundreds of kilometers, further precluded an infective process spreading across the population.

Research to date suggests that environmental conditions in the 10 days leading up to a die-off are critical and significantly associated with increased heat and humidity (*3*). The trends in climate in the region are for warmer and wetter conditions, which might have been an important factor in these recent events that have occurred irregularly over the last few decades. Immunocompetence was not thought to be a factor in the pathogenesis because the population was behaving normally, was unstressed, and was in apparent good health and body condition with large fat reserves observed postmortem. In addition, genetic analysis of the saiga antelope population shows them to be the most heterogeneous of any mammal species on record (S. Zuther, unpub. data), thus excluding inbreeding as a factor, despite the potential bottlenecking of the population in recent times.

The mechanism behind the mass die-off might be an environmentally triggered bacterial proliferation that overwhelmed the mucosal immunity of the upper respiratory and gastrointestinal tracts. This hypothesis is further supported by the observation that calves, which are unlikely to be infected with the commensal bacteria in the first couple days of life, died some hours or longer after their mothers, most likely from suckling infected milk from ill or dead mothers, activity that was observed by investigators (*3*).

In this study, HTS was used to identify pathogens that might have predisposed or contributed to the severity of the saiga antelope die-off in 2015. In previous studies, *P. multocida* type B was identified by culture, and viruses of veterinary importance (foot-and-mouth, PPR, and bluetongue viruses) were ruled out by using pathogen-specific diagnostic tests. In our study, 3 laboratories using 3 distinct HTS analytic approaches failed to identify additional pathogens. These findings, combined with clinical, necroscopic, microbiologic, and histopathologic investigations, indicate hemorrhagic septicemia caused by *P. multocida* serotype B is the proximate cause, and possibly the only cause, of this die-off. Environmental factors might have triggered nearly simultaneous bacterial proliferation and subsequent virulence in affected aggregations.

Comprehensive field monitoring and additional experimental studies of *P. multocida* infection in saiga antelope are necessary to evaluate the potential co-factors triggering the virulence of bacteria. These recurrent mass die-offs could cause extinction of saiga antelope populations in just 1 event, especially if, in future outbreaks, additional pathogens in combination with *P. multocida* affect the population.

Appendix Additional information regarding a mass die-off of saiga antelopes, Kazakhstan, 2015.

## References

[1] Mallon DP. *Saiga tatarica* ssp. In: IUCN red list of threatened species. Version 2011.2. Cambridge: International Union for Conservation of Nature Global Species Programme Red List Unit; 2011.

[2] Kock R, Grachev Y, Zhakypbayev A, Usenbayev A, Zuther S, Klimanova O, et al. G. A retrospective assessment of saiga antelope *Saiga tatarica* die-off in Western Kazakhstan 2010–2011. Saiga News. 2011;14:1–4.

[3] Kock R, Orynbayev M, Robinson S, Zuther S, Singh N, Beauvais W, et al. Saigas on the brink: multi-disciplinary analysis of the factors influencing mass mortality events. Sci Adv. 2018;4:eaao2314. 10.1126/sciadv.aao2314 PMC5777396 29376120

[4] Zuther S. The saiga antelope mass die-off in the Betpak-Dala population in May 2015. Saiga News. 2016;20:6–8.

[5] Abdrakhmanov A. Overview of the 2015 saiga mass mortality event in Kazakhstan. Presented at: Technical Workshop of the Third Meeting of Signatories of the Memorandum of Understanding Concerning Conservation, Restoration, and Sustainable Use of the Saiga Antelope (*Saiga* spp.), October 2015, Tashkent, Uzbekistan [in Russian] [cited 2018 Jan 7]. https://www.cms.int/en/document/technical-workshop-2610-overview-2015-saiga-mass-mortality-event-kazakhstan

[6] Orynbayev M, Sultankulova K, Sansyzbay A, Rystayeva R, Shorayeva K, Namet A, et al. Biological characterization of *Pasteurella multocida* present in the Saiga population. BMC Microbiol. 2019;19:37. 10.1186/s12866-019-1407-9 30744550 PMC6371526

[7] Altschul SF, Madden TL, Schäffer AA, Zhang J, Zhang Z, Miller W, et al. Gapped BLAST and PSI-BLAST: a new generation of protein database search programs. Nucleic Acids Res. 1997;25:3389–402. 10.1093/nar/25.17.3389 9254694 PMC146917

[8] Chakravorty S, Helb D, Burday M, Connell N, Alland D. A detailed analysis of 16S ribosomal RNA gene segments for the diagnosis of pathogenic bacteria. J Microbiol Methods. 2007;69:330–9. 10.1016/j.mimet.2007.02.005 17391789 PMC2562909

[9] Singh NJ, Grachev IA, Bekenov AB, Milner-Gulland EJ. Saiga antelope calving site selection is increasingly driven by human disturbance. Biol Conserv. 2010;143:1770–9. 10.1016/j.biocon.2010.04.026

[10] Milner-Gulland E, Kholodova M, Bekenov A, Bukreeva O, Grachev I, Amgalan L, et al. Dramatic declines in saiga antelope populations. Oryx. 2001;35:340–5. 10.1017/S0030605300032105

[11] Grachev Y. Results of the 2013 aerial survey in Kazakhstan. Saiga News. 2013;17:5.

[12] Orynbayev MB, Beauvais W, Sansyzbay AR, Rystaeva RA, Sultankulova KT, Kerimbaev AA, et al. Seroprevalence of infectious diseases in saiga antelope (*Saiga tatarica tatarica*) in Kazakhstan 2012-2014. Prev Vet Med. 2016;127:100–4. 10.1016/j.prevetmed.2016.03.016 27094147

[13] Bekenov AB, Pole SB, Khakhin GV, Grachev YA. Mortality from diseases and parasitic invasions. In: Sokolov VE and Zhirnov LV, editors. The saiga antelope. Moscow: Russian Academy of Sciences; 1998. p 247–52.

[14] Höper D, Mettenleiter TC, Beer M. Metagenomic approaches to identifying infectious agents. Rev Sci Tech. 2016;35:83–93. 10.20506/rst.35.1.2419 27217170

[15] Conceição-Neto N, Zeller M, Lefrère H, De Bruyn P, Beller L, Deboutte W, et al. Modular approach to customise sample preparation procedures for viral metagenomics: a reproducible protocol for virome analysis. Sci Rep. 2015;5:16532. 10.1038/srep16532 26559140 PMC4642273

[16] Yooseph S, Andrews-Pfannkoch C, Tenney A, McQuaid J, Williamson S, Thiagarajan M, et al. A metagenomic framework for the study of airborne microbial communities. PLoS One. 2013;8:e81862. 10.1371/journal.pone.0081862 24349140 PMC3859506

[17] Afshinnekoo E, Meydan C, Chowdhury S, Jaroudi D, Boyer C, Bernstein N, et al. Geospatial resolution of human and bacterial diversity with city-scale metagenomics. Cell Syst. 2015;1:72–87. 10.1016/j.cels.2015.01.001 26594662 PMC4651444

