# Supplementary material for: Mass Die-Off of Saiga Antelopes, Kazakhstan, 2015
Source: Emerg Infect Dis. 2019 Jun;25(6):1169–76. doi: 10.3201/eid2506.180990 (PMC6537709; doi:10.3201/eid2506.180990)
Supplement: Appendix — Additional information regarding a mass die-off of saiga antelopes, Kazakhstan, 2015. [file 18-0990-Techapp-s1.pdf]

Article DOI: https://doi.org/10.3201/eid2506.180990

Mass Die-Off of Saiga Antelopes, Kazakhstan, 2015

Appendix

Random Amplification–Based Sequencing Protocol

A random amplification-based sequencing protocol designed to detect both RNA and DNA based pathogens was employed at Pirbright to further investigate the blood spots. For random amplification-based sequencing, total nucleic acids were extracted by excising 1cm diameter discs of the blood spot and incubating these in 200µl of RLT buffer (with 1% β-mercaptoethanol) (Qiagen) at 56°C for 15 minutes, before supernatant was decanted off and nucleic acids extracted using the RNeasy mini kit (Qiagen). All samples were processed individually to minimise cross-contamination with periods of at least 30 minutes separating the handling of subsequent samples. Extracted nucleic acid was validated for further investigations for the presence of the highly-conserved housekeeping gene (GAPDH) using an established qualitative real-time RT-PCR assay (*1*). Total extracted nucleic acid was also tested for the presence of notifiable transboundary ruminant viral pathogens using previously established diagnostic real-time RT-PCR assays for foot-and-mouth disease virus (*2*), blue-tongue virus (*3*), peste des petits ruminants virus (*4*), capripox viruses (*5*) and epizootic haemorrhagic disease virus (*6*).

All 12 samples that were positive for the presence of GAPDH, suggestive of intact nucleic acid, were subjected to further investigations. Random amplification of nucleic acid of 6 GAPDH positive samples (samples 1–6) was performed using the SeqPlex kit (Sigma). Briefly, 3.3µL of extracted nucleic acid was subjected to random amplification using manufacturer's protocols. Amplification products were run in a 1% agarose gel at 60V for 1 hour and a 300bp band was excised and purified using a Qiagen MinElute kit (Qiagen) and eluted in 10uL. The elution was quantified using a nanodrop, qubit dsDNA BR kit (Life technologies) and Bioanalyser dsDNA kit (Agilent). Five hundred nanograms of amplicon were used to prepare

sequencing libraries using a Kapa Hyper prep kit (KapaBiosciences). Constructed libraries were purified and quantified using a Kapa Illumina library quantification kit (Kapa Biosciences). Libraries were diluted to 15pM and loaded onto a MiSeq 2x300 version 3 reagent cartridge and run on an Illumina MiSeq with a 5% PhiX spike-in control. Files were run as fastq only and transferred onto a high-performance computing cluster for further analysis.

Two separate analysis protocols were employed to interrogate the data; a k-mer based approach and a de-novo approach (*7*,*8*). Before both protocols adaptors were removed, and reads quality trimmed (using a q-score threshold of 30), with trim-galore (version 0.4.3) (*9*). For the k-mer based protocol, any reads less than 100 bases in length were removed. All remaining reads were then analyzed and classified taxonomically using the Kraken mapping software (version 0–15-β) using a local database for identification of all viruses, bacteria and archaeal genomes extracted from the NCBI refseq database (downloaded 15/04/2017). Results were visualized using Kronatools (*10*). In the case of the *de-novo* protocol, the sequencing reads from all samples were initially pooled together and then assembled using SPAdes (*11*). Only contigs with length 200 nt or more were kept, to filter out possible false positives due to short sequences. The resulting contigs were scanned against several BLAST (*12*) pre-generated databases downloaded from NCBI (*nt*, *tsa_nt*, *ref_prok_rep_genomes*, *ref_viruses_rep_genomes*, and *vector* – downloaded 15/04/2017). The results were filtered keeping only the best hits and discarding hits having <80% similarity with the query, and length <50% than the length of the query. Hits were subsequently accumulated by species. As most BLAST databases containing genomes of organisms assembled with Illumina technologies also contain the phiX genome due to it being present as positive sequencing control, we manually fished out the phiX contigs before running BLAST. The contigs for which no hit from any of the 5 BLAST databases was recorded were classified as unknown. The unknown contigs were subsequently scanned against the same BLAST databases using TBLASTX (*13*), to find similarities at protein level. Only hits with e-value $<10^{-5}$, and only the best hit per contig, were kept, to reduce the number of spurious matches. Separately, the unknown contigs were also translated using all possible open reading frames and subsequently processed with SUPERFAMILY version 1.75 (*13*) to identify potential homologies with known proteins. The raw paired fastq reads generated by this protocol and subsequent assemblies (identifiable and unknown) were submitted to the European nucleotide archive (ENA) archive under accession no. (to be confirmed).

A local contamination by an unrelated laboratory adapted avian coronavirus (Infectious Bronchitis Virus IBV M41-CK) was detected by both methods. To exclude the possibility that IBV was present in the original RNA samples, we tested remaining stocks of original RNA using an IBV-specific 5′UTR RT-qPCR (*14*), which was negative in 6/6 samples tested. IBV reads/contigs were then excluded from subsequent analysis and conclusions, as they were not considered to have any impact on the investigation.

RNA Sequencing Protocol

This protocol was applied by FLI, and RNA was extracted from blood spots on FTA cards. To this end, before extraction, from each FTA card representing one individual, five 5mm punches were ground in a 2ml tube with a 5mm stainless steel bead in 1ml Trizol (Invitrogen) using a TissueLyser (Qiagen) set at 20 Hz for 3 min. Thereafter, the tubes were spun in a standard table-top centrifuge at 13,000 rpm and the supernatant transferred to a fresh tube. Subsequently, the published protocol for the extraction of RNA was applied (*15*). In brief, the aqueous phase was mixed with ethanol (40% v/v) and this mixture transferred to a Qiagen RNeasy spin column and all further steps, including the optional on-column DNase treatment, carried out according to the manufacturer's instructions. The extracted RNA was quantified using a Nanodrop ND1000 instrument (Peqlab, Erlangen, Germany), and 500 ng were used for cDNA synthesis and library preparation as described (*16*). Briefly, after the addition of random hexamer primers, the RNA was denatured at 95°C for 2 min, immediately followed by snap-freezing. This RNA-primer mix was used as input for reverse transcription and second strand synthesis with the cDNA synthesis system kit (Roche, Mannheim, Germany). The obtained double-stranded cDNA was fragmented to a peak size of approx. 500 bp using the M220 Focused-ultrasonicator (Covaris, Brighton, United Kingdom) and used as input for library preparation with a GeneRead DNA Library L Core Kit (Qiagen) and Ion Xpress Barcode Adapters (Life Technologies, Darmstadt, Germany). After quality control with an Agilent Bioanalyzer 2100 DNA HS kit (Agilent, Waldbronn, Germany) and quantification with the KAPA Library Quantification Kit - Ion Torrent Universal (Roche), the resulting libraries (libraries lib01416, lib01417, lib01418, lib01419; corresponding to samples 2, 5, 8, 11) were sequenced using the Ion Torrent PGM (Life Technologies) with 400 bp HiQ reagents following the manufacturer's instructions. The obtained datasets were analyzed using the software pipeline

RIEMS (*17*). In addition, the datasets were mapped along the available *P. multocida* genome sequence (NC_002663.1) using the Roche/454 software suite (v3.0; Roche) and the generated contigs analyzed using BLASTX (v2.2.26+) (*12*). The raw reads generated by this protocol were submitted to the ENA archive under accession no. PRJEB28164.

16S Metagenomic Sequencing Protocol

This protocol was applied by IMV, and microbial DNA was extracted from lung and kidney tissues separately using Trizol Reagent (Thermo Fisher Scientific, USA) according to manufacturer's recommendations.

Library preparation was conducted according to Illumina 16S Metagenomic Sequencing Library Preparation Workflow (Illumina, USA). This protocol combined with a benchtop sequencing system, on-board primary analysis, and secondary analysis using MiSeq Reporter or BaseSpace, provides a comprehensive workflow for 16S rRNA amplicon sequencing. Briefly, the 16S Amplicon PCR forward and reverse primers (recommended by Illumina) were used to amplify the bacterial V3 and V4 regions (with ≈460 bp length). PCR products were purified using magnetic AMPure XP beads (Beckman Coulter, USA) to remove free primers and primer dimers to avoid interference with the sequencing process. Then amplification of the V3 and V4 region using a limited cycle PCR with simultaneous addition of Nextera XT (Illumina, USA) sequencing adapters and dual indexed barcodes to the amplicon target was conducted. AMPure XP beads were used to clean up the final library before quantification on Qubit 2.0 spectrophotometer (Thermo Fisher Scientific, USA). Fragments were visualized on an agarose gel to check quality and average nucleotide length.

Sequencing was performed on an Illumina MiSeq using Illumina v.3 reagent kit with a 7.5% PhiX (Illumina, USA) spike-in control. Data were analyzed locally by on-board MiSeq Reporter software (Illumina, USA). Taxonomic classification was performed using the Greengenes database showing genus or species level classification in a graphical and table format. The 16S sequencing metagenome dataset file was submitted to Genbank under accession no. PRJNA486600.

References

1. King DP, Burman A, Gold S, Shaw AE, Jackson T, Ferris NP. Integrin sub-unit expression in cell cultures used for the diagnosis of foot-and-mouth disease. Vet Immunol Immunopathol. 2011;140:259–65. PubMed http://dx.doi.org/10.1016/j.vetimm.2011.01.008

2. Callahan JD, Brown F, Osorio FA, Sur JH, Kramer E, Long GW, et al. Use of a portable real-time reverse transcriptase-polymerase chain reaction assay for rapid detection of foot-and-mouth disease virus. J Am Vet Med Assoc. 2002;220:1636–42. PubMed http://dx.doi.org/10.2460/javma.2002.220.1636

3. Hofmann M, Griot C, Chaignat V, Perler L, Thür B. Bluetongue disease reaches Switzerland [in German]. Schweiz Arch Tierheilkd. 2008;150:49–56. PubMed http://dx.doi.org/10.1024/0036-7281.150.2.49

4. Batten CA, Banyard AC, King DP, Henstock MR, Edwards L, Sanders A, et al. A real time RT-PCR assay for the specific detection of Peste des petits ruminants virus. J Virol Methods. 2011;171:401–4. 10.1016/j.jviromet.2010.11.022 PubMed http://dx.doi.org/10.1016/j.jviromet.2010.11.022

5. Bowden TR, Babiuk SL, Parkyn GR, Copps JS, Boyle DB. Capripoxvirus tissue tropism and shedding: A quantitative study in experimentally infected sheep and goats. Virology. 2008;371:380–93. PubMed http://dx.doi.org/10.1016/j.virol.2007.10.002

6. Maan NS, Maan S, Potgieter AC, Wright IM, Belaganahalli M, Mertens PPC. Development of real-time RT-PCR assays for detection and typing of epizootic haemorrhagic disease virus. Transbound Emerg Dis. 2017;64:1120–32. 10.1111/tbed.12477 PubMed http://dx.doi.org/10.1111/tbed.12477

7. Wood DE, Salzberg SL. Kraken: ultrafast metagenomic sequence classification using exact alignments. Genome Biol. 2014;15:R46. 10.1186/gb-2014-15-3-r46 PubMed http://dx.doi.org/10.1186/gb-2014-15-3-r46

8. Marco-Sola S, Sammeth M, Guigó R, Ribeca P. The GEM mapper: fast, accurate and versatile alignment by filtration. Nat Methods. 2012;9:1185–8. PubMed http://dx.doi.org/10.1038/nmeth.2221

9. Babraham Bioinformatics. Trim Galore [cited 15 April 2017]. http://www.bioinformatics.babraham.ac.uk/projects/trim_galore

10. Ondov B. KronaTools 2.7 [cited 2017 Apr 13]. https://github.com/marbl/Krona/wiki/KronaTools

11. Bankevich A, Nurk S, Antipov D, Guevich AA, Dvorkin M, Kulikov AS, et al. SPAdes: a new genome assembly algorithm and its applications to single-cell sequencing. J Comput Biol. 2012;19:455–77. PubMed http://dx.doi.org/10.1089/cmb.2012.0021

12. Altschul SF, Madden TL, Schäffer AA, Zhang J, Zhang Z, Miller W, et al. Gapped BLAST and PSI-BLAST: a new generation of protein database search programs. Nucleic Acids Res. 1997;25:3389–402. PubMed http://dx.doi.org/10.1093/nar/25.17.3389

13. Wilson D, Pethica R, Zhou Y, Talbot C, Vogel C, Madera M, et al. SUPERFAMILY—comparative genomics, datamining and sophisticated visualisation. Nucleic Acids Res. 2009;37:D380–6. PubMed http://dx.doi.org/10.1093/nar/gkn762

14. Callison SA, Hilt DA, Boynton TO, Sample BF, Robison R, Swayne DE, et al. Development and evaluation of a real-time Taqman RT-PCR assay for the detection of infectious bronchitis virus from infected chickens. J Virol Methods. 2006;138:60–5. PubMed http://dx.doi.org/10.1016/j.jviromet.2006.07.018

15. Höper D, Hoffmann B, Beer M. A comprehensive deep sequencing strategy for full-length genomes of influenza A. PLoS One. 2011;6:e19075. PubMed http://dx.doi.org/10.1371/journal.pone.0019075

16. Juozapaitis M, Aguiar Moreira É, Mena I, Giese S, Riegger D, Pohlmann A, et al. An infectious bat-derived chimeric influenza virus harbouring the entry machinery of an influenza A virus. Nat Commun. 2014;5:4448. PubMed http://dx.doi.org/10.1038/ncomms5448

17. Scheuch M, Höper D, Beer M. RIEMS: a software pipeline for sensitive and comprehensive taxonomic classification of reads from metagenomics datasets. BMC Bioinformatics. 2015;16:69. PubMed http://dx.doi.org/10.1186/s12859-015-0503-6

Appendix Table 1. Viruses and bacteria identified with the Kraken analysis protocol from the data produced using the random amplification meta-transcriptomic protocol. Numbers in parentheses represent % of total reads

Organism	Sample 1	Sample 2	Sample 3	Sample 4	Sample 5	Sample 6
Viruses						
Enterobacteria phage phiX174 sensu lato *	18742 (7.79)	46303 (4.9)	16755 (4.8)	22694 (4.85)	19518 (5.69)	13204 (4.77)
Infectious Bronchitis virus (M41-CK) *	18 (0.01)	8394 (0.55)	6 (0)	52 (0.01)	19 (0.01)	36 (0.01)
Enterobacteria phage M13	2 (0)	–	–	–	–	–
Enterobacteria phage ID2 Moscow/ID/2001	–	3 (0)	–	–	–	–
Haemophilus phage SuMu	41 (0.02)	48 (0.01)	22 (0.01)	40 (0.01)	45 (0.01)	32 (0.01)
Primula malacoides virus China/Mar2007	1 (0)	34 (0)	1 (0)	104 (0.02)	11 (0)	9 (0.01)
Hyposoter fugitivus ichnovirus	1 (0)	–	–	–	–	–
Torque teno midi virus 2	–	3 (0)	1 (0)	2 (0)	–	–
Elephantid herpesvirus 1	1 (0)	2 (0)	–	6 (0)	1 (0)	–
Parvovirus NIH-CQV	–	2 (0)	–	–	2 (0)	–
Jingmen tick virus	–	10 (0)	–	4 (0)	2 (0)	2 (0)
Solenopsis invicta virus 3	–	3 (0)	–	–	–	–
Carp picornavirus 1	–	6 (0)	–	–	3 (0)	3 (0)
Eel picornavirus 1	–	1 (0)	–	–	–	–
Mouse astrovirus M-52/USA/2008	1 (0)	4 (0)	–	–	–	1 (0)
Spring beauty latent virus	–	2 (0)	–	–	1 (0)	–
Hepatitis C virus	–	1 (0)	–	2 (0)	1 (0)	–
Dolphin morbillivirus	–	4 (0)	–	–	–	–
Dickeya phage RC-2014	–	3 (0)	2 (0)	6 (0)	1 (0)	–
Cynomolgus macaque cytomegalovirus (Ottawa)	–	1 (0)	–	–	–	–
Grapevine Syrah virus 1					1 (0)	–
Bacillus phage SPO1	–	–	2 (0)	–	1 (0)	2 (0)
Cercopithecine herpesvirus 2	–	2 (0)	–	–	–	1 (0)
Ictalurid herpesvirus 1	–	2 (0)	–	–	–	–
Cyprinid herpesvirus 1	–	2 (0)	–	–	–	–
Pandoravirus salinus	–	1 (0)	2 (0)	4 (0)	–	–
Hyposoter fugitivus ichnovirus	–	2 (0)	–	–	1 (0)	–
Glypta fumiferanae ichnovirus	–	–	1 (0)	–	–	–
Cotesia congregata bracovirus	–	1 (0)	–	–	–	–
Maruca vitrata nucleopolyhedrovirus	–	1 (0)	–	–	–	–
Orgyia leucostigma NPV	–	–	–	2 (0)	–	–
Phaeocystis globosa virus	–	1 (0)	–	4 (0)	2 (0)	–
Hop trefoil cryptic virus 2	–	1 (0)	–	–	1 (0)	–
Y73 sarcoma virus	–	1 (0)	–	–	–	–
Solenopsis invicta virus 3	–	–	1 (0)	–	–	–
Red clover cryptic virus 2	–	–	1 (0)	–	–	–
Dulcamara mottle virus	–	–	–	–	–	1 (0)
Bacteria						
Unclassified	14019 (58.34)	810950 (73.3)	219326 (62.8)	266912 (57.06)	254164 (54.02)	205048 (54.81)
Pasteurella multocida	52771 (28.56)	121145 (14.4)	62719 (23.23)	88348 (24.27)	91558 (25.45)	71673 (24.99)
Alteromonas macleodii str. 'Ionian Sea U8'	1366 (0.57)	2724 (0.25)	46 (0.01)	980 (0.21)	4350 (0.92)	7737 (2.03)
Achromobacter xylosoxidans	2 (0)	13247 (1.2)	22 (0.01)	82 (0.02)	3423 (0.72)	6036 (1.61)
Dickeya dadantii Ech703	69 (0.03)	95 (0.01)	103 (0.03)	78 (0.02)	76 (0.01)	42 (0.02)
Haemophilus spp.	23 (0.01)	95 (0.01)	43 (0.01)	72 (0.02)	76 (0.01)	42 (0.02)
Klebsiella variicola At-22	11 (0)	58 (0.01)	16 (0)	54 (0.01)	16 (0)	14 (0)
Mannheima haemolytica	10 (0)	13 (0)	12 (0)	18 (0)	22 (0)	7 (0)
Mycoplasma spp.	13 (0.01)	312 (0.02)	32 (0.01)	42 (0.01)	29 (0.01)	13 (0)
Rickettsia africae ESF-5	1 (0)	5 (0)	1 (0)	18 (0)	29 (0)	33 (0.01)
Campylobacter spp. (C.jejuni subsp.)	3 (0)	39 (0)	25 (0.01)	18 (0.01)	5 (0)	8 (0)
Aggregatibacter	8 (0)	6 (0)	7 (0)	18 (0)	3 (0)	4 (0)
Candidatus Riesia pediculicola USDA	2 (0)	13 (0)	9 (0)	8 (0)	3 (0)	4 (0)
Vibrio spp. (inc. V.Cholerae)	2 (0)	23 (0)	13 (0)	14 (0)	11 (0)	8 (0)
Dichelobacter nodosus VC51703A	80 (0.03)	6 (0)	3 (0)	2 (0)	1 (0)	1 (0)
Histophilus somni	1 (0)	14 (0)	5 (0)	6 (0)	3 (0)	1 (0)

*Reads aligning to PhiX and avian Coronavirus Infectious Bronchitis Virus M41-CK (IBV) were present in the final datasets. These were attributed to the positive sequencing control (PhiX) and a local contamination by a lab adapted strain of IBV, respectively (the presence of IBV in the original samples was excluded by RT-qPCR, as explained in the methods).

Appendix Table 2. Results from the *de-novo* assembly protocol applied to samples sequenced using the random amplification meta-transcriptomic protocol. The attribution as determined by the best BLASTN hit; the number of contigs with the same attribution; and their total length, are listed in columns 4, 2, and 3, respectively. In column 1 the read area (equivalent to the contig length times the average read coverage) is listed.

Area (length*coverage)	Contigs	Total length	Attribution
21640705	6013	2025938	Pasteurella multocida
12470305	1	5421	Enterobacteria phage phiX174 sensu lato
462023	602	108406	UNKNOWN SEQUENCE
397727	47	26577	Infectious bronchitis virus
351922	235	43608	Gallus gallus (chicken)
103710	353	55287	Ovis canadensis canadensis
71394	5	590	*Escherichia coli*
53466	1	230	uncultured Pasteurella sp.
49238	3	397	Lasius turcicus
29395	2	293	Cloning vector lambda EMBL3 SP6/T7, left arm
29395	2	293	Enterobacteria phage HK630
21658	5	2901	Antidorcas marsupialis (springbok)
12173	9	2176	Ovis aries musimon (mouflon)
11830	29	5197	Capra hircus (goat)
5984	16	2081	Ovis aries (sheep)
5973	2	531	Antilope cervicapra (blackbuck)
4833	4	967	Nanger dama (Dama gazelle)
4271	6	2113	Eudorcas thomsonii (Thomson's gazelle)
3527	14	2579	Bubalus bubalis (water buffalo)
2687	8	1544	Saiga tatarica
2331	8	1020	Numida meleagris (helmeted guineafowl)
2119	24	6912	Bos taurus (cattle)
1779	9	1684	Bos indicus
1246	1	600	Madoqua kirkii (Kirk's dik-dik)
1033	4	513	Apteryx australis mantelli
893	4	530	Meleagris gallopavo (turkey)
854	1	330	uncultured bacterium
729	3	361	Sus scrofa (pig)
484	1	249	Gazella leptoceros (Rhim gazelle)
446	1	207	Phascolarctos cinereus (koala)
446	3	303	Odocoileus virginianus texanus
391	2	317	Pantholops hodgsonii (chiru)
376	1	313	Odocoileus hemionus (mule deer)
372	2	255	Chinchilla lanigera (long-tailed chinchilla)
146	1	206	Onchocerca flexuosa
0	1	256	Schmidtea mediterranea

Appendix Table 3. Results from the *de-novo* assembly protocol applied to samples sequenced using the random amplification meta-transcriptomic protocol. TBLASTX results for the contigs unassigned by BLASTN (*Unknown sequence*).

Area (length*coverage)	Contigs	Total length	Attribution
121760	70	33252	Pasteurella multocida
17650	69	10509	Ovis canadensis canadensis
15395	10	2379	Eudorcas thomsonii (Thomson's gazelle)
7154	8	2158	Antidorcas marsupialis (springbok)
4491	1	566	Gazella leptoceros (Rhim gazelle)
3574	1	486	Haemophilus influenzae
3020	2	999	Infectious bronchitis virus
2425	2	311	Nanger dama (Dama gazelle)
2019	12	2547	Gallus gallus (chicken)
1494	2	869	Avibacterium paragallinarum JF4211
1302	5	651	Apteryx australis mantelli
1102	1	213	Pasteurella bettyae CCUG 2042
1087	3	562	Meleagris gallopavo (turkey)
1067	1	241	Avibacterium paragallinarum
1015	4	663	Bubalus bubalis (water buffalo)
966	6	1133	Capra hircus (goat)
942	1	270	Raphicerus sharpei (Sharpe's grysbok)
756	1	180	Bos indicus
726	1	253	Coturnix japonica (Japanese quail)
701	1	209	Actinobacillus succinogenes 130Z
552	3	512	Bos taurus (cattle)
543	1	173	Nanger granti (Grant's gazelle)
542	1	313	Haemophilus parasuis SH0165
540	2	347	Pseudorca crassidens (false killer whale)
519	1	593	Haemophilus somnus 129PT
518	1	295	Ficedula albicollis (collared flycatcher)
482	1	237	Haemophilus influenzae 2019
476	2	238	Ovis aries (sheep)
445	1	171	Odocoileus virginianus texanus
444	3	646	Antilope cervicapra (blackbuck)
312	1	133	Numida meleagris (helmeted guineafowl)
292	1	184	Bibersteinia trehalosi USDA-ARS-USMARC-189
248	1	124	groundwater metagenome
233	1	222	Catellicoccus marimammalium M35/04/3
89	1	218	Lepidonotothen nudifrons (yellowfin notie)ID

Appendix Table 4. Results from the *de-novo* assembly protocol applied to samples sequenced using the random amplification meta-transcriptomic protocol. SUPERFAMILY hits for the contigs unassigned by BLASTN (*Unknown sequence*). Columns list contig name with frame and peptide counter appended; peptide region matched; SUPERFAMILY score; and SUPERFAMILY attribution.

Contig name	Peptide region	Score	Attribution
NODE_1869_length_403_cov_0.699752_g1587_i0_6_1	23468	2.59E-12	Actin/HSP70
NODE_477_length_778_cov_3.95861_g348_i0_5_1	33512	5.50E-23	FKBP immunophilin/proline isomerase
NODE_477_length_778_cov_3.95861_g348_i0_5_1	91–127	5.49E-07	TF C-terminus
NODE_477_length_778_cov_3.95861_g348_i0_6_2	27–136	1.31E-25	TF C-terminus
NODE_570_length_733_cov_1.83356_g423_i0_5_1	13–101	1.70E-14	Band 7/SPFH domain
NODE_570_length_733_cov_1.83356_g423_i0_6_2	30–83	1.16E-08	Band 7/SPFH domain
NODE_579_length_724_cov_6.30041_g430_i0_1_1	6–103	3.17E-30	Pseudouridine synthase II TruB
NODE_579_length_724_cov_6.30041_g430_i0_2_3	34–97	2.88E-12	Pseudouridine synthase II TruB
NODE_579_length_724_cov_6.30041_g430_i0_2_3	101–139	4.37E-03	PUA domain
NODE_2811_length_299_cov_5.0903_g2487_i0_6_1	12510	5.10E-04	NadC C-terminal domain-like
NODE_2367_length_343_cov_2.73105_g2045_i0_5_1	15950	1.88E-03	Porin chaperone SurA
NODE_2430_length_336_cov_7.68899_g2106_i0_1_1	43160	1.32E-03	Rubredoxin
NODE_2431_length_336_cov_3.47619_g2107_i0_2_1	23346	3.79E-14	LemA-like
NODE_2461_length_334_cov_2.71307_g2137_i0_3_1	18872	5.56E-10	GlnE-like domain
NODE_284_length_936_cov_3.66346_g202_i0_5_1	80–142	6.10E-21	FtsK C-terminal domain-like
NODE_284_length_936_cov_3.66346_g202_i0_5_2	32–148	1.96E-37	Outer-membrane lipoproteins carrier protein LolA
NODE_5168_length_140_cov_2_g4844_i0_1_2	43922	7.78E-03	B-box zinc binding domain
NODE_1234_length_521_cov_1.29175_g1000_i0_2_1	28430	5.30E-19	Lambda integrase-like
NODE_1287_length_506_cov_4.32016_g1049_i0_4_2	36–63	1.05E-05	Fumarate reductase/Succinate dehydogenase iron-sulfur protein
NODE_1287_length_506_cov_4.32016_g1049_i0_5_1	35247	4.71E-11	Fumarate reductase/Succinate dehydogenase iron-sulfur protein
NODE_4140_length_209_cov_3.35407_g3816_i0_6_1	19603	9.84E-07	Phage repressors
NODE_1037_length_567_cov_3.37449_g824_i0_2_1	34912	2.09E-06	HlyD-like secretion proteins
NODE_1104_length_550_cov_1.47091_g885_i0_4_2	22–66	3.27E-04	Mitotic arrest deficient-like 1
NODE_1422_length_476_cov_5.28361_g1175_i0_6_1	40–70	8.76E-05	Multidrug efflux transporter AcrB TolC docking domain
NODE_1431_length_474_cov_1.90443_g1183_i0_4_1	24351	1.37E-12	GHMP Kinase
NODE_1431_length_474_cov_1.90443_g1183_i0_6_2	16–75	4.02E-13	GHMP Kinase
NODE_1457_length_470_cov_0.501064_g1109_i1_5_1	32448	6.97E-15	glucose-1-phosphate thymidylyltransferase
NODE_1520_length_457_cov_2.34792_g1268_i0_1_3	45323	9.61E-03	DinB-like
NODE_1520_length_457_cov_2.34792_g1268_i0_2_1	11–102	8.50E-10	Kelch motif
NODE_88_length_1421_cov_2.96129_g36_i0_2_7	27150	8.89E-16	Trp repressor
NODE_217_length_1020_cov_3.88725_g142_i0_4_2	19725	1.50E-11	Nitrogenase iron protein-like
NODE_5735_length_120_cov_2_g5411_i0_4_1	12451	4.19E-05	FAD-dependent thiol oxidase
NODE_5526_length_128_cov_2_g5202_i0_1_1	46419	3.36E-03	B-box zinc binding domain
NODE_5657_length_123_cov_2_g5333_i0_2_1	43983	7.45E-03	TM1622-like
NODE_5665_length_123_cov_2_g5341_i0_1_1	12086	3.57E-03	Variant RING domain
NODE_5682_length_122_cov_2_g5358_i0_1_1	13119	5.49E-03	DNA binding domain of intron-encoded endonucleases
NODE_5427_length_131_cov_1.67939_g5103_i0_2_1	45717	9.16E-03	Myotoxin
NODE_6013_length_108_cov_2.32407_g5689_i0_2_1	43466	8.89E-03	HIT zinc finger
NODE_188_length_1067_cov_2.89972_g117_i0_1_2	46–215	2.88E-47	Phosphoribosylpyrophosphate synthetase-like
NODE_188_length_1067_cov_2.89972_g117_i0_2_1	27668	4.47E-22	Phosphoribosylpyrophosphate synthetase-like
NODE_188_length_1067_cov_2.89972_g117_i0_5_3	46023	1.57E-03	Regulatory protein AraC
NODE_2577_length_322_cov_1.49596_g2253_i0_1_1	24716	1.06E-10	GHMP Kinase
NODE_1552_length_451_cov_2.82927_g1295_i0_5_1	25538	1.47E-10	Decarboxylase
NODE_5795_length_117_cov_1.46154_g5471_i0_6_1	13820	8.26E-05	GpdQ-like
NODE_5279_length_136_cov_2_g4955_i0_6_1	44256	4.38E-03	Aspartate/glutamate racemase
NODE_1577_length_446_cov_0.946188_g1320_i0_5_1	30621	7.33E-05	Acetyl-CoA synthetase-like
NODE_1577_length_446_cov_0.946188_g1320_i0_6_3	19–70	2.75E-09	Acetyl-CoA synthetase-like
NODE_1670_length_432_cov_1.3287_g1408_i0_5_2	19–67	9.61E-10	RNase P protein
NODE_1706_length_426_cov_15.2986_g1442_i0_1_1	20486	3.14E-15	N-acetylmuramoyl-L-alanine amidase-like
NODE_1706_length_426_cov_15.2986_g1442_i0_6_2	45078	9.16E-03	PMP inhibitors
NODE_1706_length_426_cov_15.2986_g1442_i0_6_3	16–68	5.72E-07	Exostosin
NODE_1754_length_419_cov_0.699284_g1487_i0_2_1	28734	9.27E-18	Formate dehydrogenase/DMSO reductase
NODE_1754_length_419_cov_0.699284_g1487_i0_6_1	26634	7.84E-08	Cold shock DNA binding domain-like
NODE_6253_length_99_cov_2_g5929_i0_3_1	47150	7.54E-03	Alcohol dehydrogenase-like

NODE_6276_length_98_cov_0_g5952_i0_4_1	47119	4.64E-03	RING finger domain
NODE_4661_length_185_cov_1.33514_g4337_i0_3_1	20821	9.73E-05	Saposin B
NODE_4708_length_181_cov_10.3481_g4384_i0_6_1	19815	4.12E-09	Lambda integrase-like
NODE_2774_length_303_cov_1.51155_g2450_i0_4_1	24–63	7.55E-06	Extended AAA-ATPase domain
NODE_695_length_676_cov_0.761834_g529_i0_6_2	28–101	1.84E-10	Extended AAA-ATPase domain
NODE_797_length_639_cov_2.37715_g617_i0_1_1	4–103	5.90E-32	Phosphoribosyltransferases (PRTases)
NODE_797_length_639_cov_2.37715_g617_i0_2_2	66–131	4.85E-07	Phosphoribosyltransferases (PRTases)
NODE_812_length_633_cov_5.69217_g632_i0_4_3	6–102	1.23E-23	Arylsulfatase
NODE_861_length_616_cov_4.54708_g673_i0_2_4	25263	4.47E-16	Leukotriene A4 hydrolase catalytic domain
NODE_1422_length_476_cov_5.28361_g1175_i0_4_3	25–80	4.84E-10	Multidrug efflux transporter AcrB TolC docking domain
NODE_2887_length_293_cov_4.83276_g2563_i0_4_1	32690	3.40E-12	DAK1
NODE_3070_length_279_cov_2.09498_g2746_i0_5_1	12236	2.09E-06	Inositol monophosphatase/fructose-1
NODE_3070_length_279_cov_2.09498_g2746_i0_5_2	19511	2.96E-06	Inositol monophosphatase/fructose-1
NODE_3257_length_265_cov_1.22642_g2933_i0_5_1	46539	3.01E-03	Chorismate synthase
NODE_592_length_718_cov_7.62535_g440_i0_5_2	39–138	1.37E-32	Phosphate binding protein-like
NODE_596_length_717_cov_1.13529_g444_i0_4_1	8–167	6.00E-24	Extended AAA-ATPase domain
NODE_217_length_1020_cov_3.88725_g142_i0_5_3	97–265	1.09E-32	GABA-aminotransferase-like
NODE_217_length_1020_cov_3.88725_g142_i0_5_3	27–104	3.32E-19	RecA protein-like (ATPase-domain)
NODE_222_length_1013_cov_6.8924_g147_i0_4_1	5–282	7.06E-88	Aconitase iron-sulfur domain
NODE_255_length_976_cov_3.78586_g179_i0_5_2	69–202	2.05E-27	Glycerol kinase
NODE_255_length_976_cov_3.78586_g179_i0_6_1	43–139	3.04E-15	Glycerol kinase
NODE_284_length_936_cov_3.66346_g202_i0_1_3	12206	3.79E-03	Interleukin 8-like chemokines
NODE_3601_length_241_cov_4.42739_g3277_i0_3_1	25812	1.29E-04	Lambda integrase-like
NODE_877_length_614_cov_0.467427_g684_i0_5_3	15827	1.96E-06	ABC transporter transmembrane region
NODE_877_length_614_cov_0.467427_g684_i0_6_1	16834	7.33E-04	Neurotransmitter-gated ion-channel transmembrane pore
NODE_914_length_601_cov_10.9867_g715_i0_1_1	9–189	2.43E-26	TrmB-like
NODE_621_length_705_cov_0.92766_g466_i0_4_1	20302	1.98E-15	Extended AAA-ATPase domain
NODE_621_length_705_cov_0.92766_g466_i0_4_2	18–91	3.45E-04	Extended AAA-ATPase domain
NODE_631_length_700_cov_4.50571_g474_i0_2_4	27791	6.93E-20	UDP N-acetylglucosamine acyltransferase
NODE_631_length_700_cov_4.50571_g474_i0_3_1	8–141	1.05E-26	FabZ-like
NODE_632_length_700_cov_2.6_g475_i0_2_2	31–142	3.21E-20	Phosphate binding protein-like
NODE_693_length_676_cov_3.63757_g527_i0_5_3	25–84	2.39E-09	Thioltransferase
NODE_693_length_676_cov_3.63757_g527_i0_6_1	5–125	9.39E-19	Glutathione peroxidase-like

*Reads aligning to PhiX and avian Coronavirus Infectious Bronchitis Virus M41-CK (IBV) were present in the final datasets. These were attributed to the positive sequencing control (PhiX) and a local contamination by a lab adapted strain of IBV, respectively (the presence of IBV in the original samples was excluded by RT-qPCR, as explained in the methods).

Appendix Table 5. Summary of the most relevant results obtained by random primed cDNA shotgun sequencing

Organism	Sample 2	Sample 5	Sample 8	Sample 11
Total number reads	411,640	376,210	372,387	354,958
Number high quality reads	398,854	365,417	360,828	344,063
Number classified reads	397,078	363,897	359,202	342,569
Number unclassified reads	1,776	1,520	1,626	1,494
Number host reads	64,618	4,770	3,414	4,784
Percentage host reads	16.2	1.3	0.9	1.4
Number Pasteurellaceae reads	317,009	345,893	339,484	324,770
Percentage Pasteurellaceae reads	79.5	94.7	94.1	94.4
Number P. multocida reads	310,837	341,845	334,221	319,905
Percentage P. multocida reads	77.9	93.5	92.6	93.0

Appendix Table 6. Total species-level taxonomic categories identified. The table shows the top 8 of 68 classifications

Classification	Number of Reads	% Total Reads
Pasteurella multocida	6,907	48.32%
Unclassified at Species level	4,990	34.91%
Pasteurellaceae	1,536	10.75%
Pasteurella pneumotropica	580	4.06%
Mannheimia caviae	78	0.55%
Serratia entomophila	17	0.12%
Bacillus horneckiae	16	0.11%
Vagococcus teuberi	13	0.09%
